# Real-world longitudinal assessment of anifrolumab in patients with systemic lupus erythematosus: clinical outcomes, safety and modulation of cytokines and neutrophil activity

**DOI:** 10.1136/rmdopen-2026-006806

**Published:** 2026-06-28

**Authors:** Alp Temiz, Rita Noversa de Sousa, Janina Schoen, Clara Reichardt, Kathrin Standfest, Andreas Wirsching, Melanie Hagen, Giulia Corte, Marco Muñoz Becerra, Koray Tascilar, Axel J Hueber, Georg Schett, Luis E Muñoz, Filippo Fagni

**Affiliations:** 1 Department of Medicine 3—Rheumatology and Immunology, Friedrich-Alexander-Universität Erlangen-Nürnberg and Universitätsklinikum Erlangen, Erlangen, Germany; 2 Deutsches Zentrum Immuntherapie (DZI), Friedrich-Alexander-Universität Erlangen-Nürnberg and Universitätsklinikum Erlangen, Erlangen, Germany; 3 Serviço de Medicina Interna, Hospital Pedro Hispano, Unidade Local de Saúde de Matosinhos, Matosinhos, Portugal; 4 Division of Rheumatology, Klinikum Nürnberg, Paracelsus Medical University, Nürnberg, Germany

**Keywords:** Systemic Lupus Erythematosus, Disease Activity, Biological Therapy, Cytokines, Chemokines

## Abstract

**Objectives:**

To assess clinical effectiveness, safety and in vivo effects on cytokines, chemokines and low-density neutrophils (LDNs) in patients with active systemic lupus erythematosus (SLE) treated with anifrolumab over a 12-month real-world follow-up.

**Methods:**

We established a longitudinal, multicentre, observational cohort of adult patients with active SLE receiving anifrolumab 300 mg intravenously every 4 weeks. Patients with active lupus nephritis or central nervous system involvement were excluded. Clinical and laboratory measures were recorded at baseline and at months 3, 6, 9 and 12. Chemokines, cytokines, LDNs and neutrophil extracellular trap degradation products were assessed at the respective time points. Longitudinal changes were analysed using generalised additive models with patient-level random intercepts.

**Results:**

Twenty patients were recruited. Baseline mean Systemic Lupus Erythematosus Disease Activity Index 2000 (SLEDAI-2K) was 10.9±5.0, with frequent musculoskeletal, immunological and cutaneous involvement. Over 12 months, mean SLEDAI-2K declined to 3.1 (95% CI 1.1 to 5.1; p<0.001). SLE Responder Index-4 response rates were 64.7% at month 12 in intention-to-treat analyses with non-responder imputation. By month 12, 86% of patients reached Lupus Low Disease Activity State and 50% achieved Definition of Remission in SLE remission. Physician Global Assessment improved from 1.6 to 0.2 (95% CI 0.0 to 0.5; p<0.001). Daily prednisolone equivalents were significantly reduced (p=0.036); a dose ≤5 mg/day was achieved in 11/13 patients receiving glucocorticoids at baseline, including three who discontinued glucocorticoids. Drug persistence at 12 months was 65%. Fifty-five adverse events were recorded, of which four were serious. Anifrolumab significantly reduced circulating LDNs (p=0.032), interferon-driven chemokines and inflammatory cytokines.

**Conclusion:**

This real-world cohort provides clinical and mechanistic evidence that anifrolumab improves clinical outcomes, enables glucocorticoid tapering and reduces LDNs as well as interferon-driven chemokines in patients with active SLE. Safety and drug persistence were consistent with clinical trial data.

**Trial registration number:**

DRKS00024360.

**WHAT IS ALREADY KNOWN ON THIS TOPIC**

- Type I interferon signalling is a central pathogenic pathway in systemic lupus erythematosus (SLE), and anifrolumab has demonstrated clinical efficacy and glucocorticoid-sparing effects in phase II and III trials; however, prospective real-world data on long-term effectiveness, safety and in vivo immunological effects, particularly on neutrophil subsets and interferon-driven mediators, remain limited.

**WHAT THIS STUDY ADDS**

- In a 12-month multicentre real-world cohort, anifrolumab led to marked and sustained reductions in global and domain-specific disease activity, high rates of SLE Responder Index-4, Lupus Low Disease Activity State and Definition of Remission in SLE attainment and substantial glucocorticoid tapering.
- Clinical improvement occurred without significant changes in antidouble-stranded DNA titres or complement levels, consistent with anifrolumab’s interferon-targeted rather than B-cell-targeted mechanism.
- The study provides mechanistic evidence that anifrolumab reduces circulating low-density neutrophils and broadly downregulates interferon-associated chemokines and inflammatory cytokines in vivo.

**HOW THIS STUDY MIGHT AFFECT RESEARCH, PRACTICE OR POLICY**

- These findings support the effectiveness and acceptable safety of anifrolumab in routine clinical practice and highlight its role as a targeted option for interferon-driven SLE phenotypes.
- The combined clinical and immunological data could inform patient selection, monitoring strategies and future research on biomarkers and long-term outcomes under interferon blockade.

## Introduction

 Systemic lupus erythematosus (SLE) is a chronic systemic autoimmune disease with a prevalence of approximately 0.1% in the general population, predominantly affecting young women.^1^ SLE is characterised by dysregulation of both the innate and adaptive immune system with deposition of autoantibodies and complement in the affected tissues, leading to cell-complex-mediated and immune-complex-mediated organ injury.^1 2^ Despite advances in B-cell-targeted and T-cell-targeted therapies, a large proportion of patients with SLE continue to experience persistent disease activity, with an estimated three out of four failing to achieve durable remission.^3 4^ This situation highlights the importance of additional pathogenic pathways, particularly involving the innate immune system. Loss of tolerance to nuclear antigens leads to aberrant immune activation, autoreactive B-cell and T-cell generation and production of antibodies against DNA and nuclear proteins. Importantly, immune complex formation and exposure of nucleic acids triggers type I interferon (IFN-I) production by plasmacytoid dendritic cells and other immune cells, which in turn amplifies autoantibody production and antigen presentation.^5 6^ Transcriptomic studies have identified an IFN signature in a distinct subset of patients, and serum IFN-I levels correlate with disease activity.^7 8^ Low-density neutrophils (LDNs) are a subset of neutrophils with enhanced inflammatory potential and proposed to participate in the pathogenesis of SLE by spontaneously releasing neutrophil extracellular traps (NETs).^9 10^ The latter are able to trigger toll-like receptors on plasmacytoid dendritic cells, promoting the production of interferon alpha (IFNα), which in turn primes neutrophils to release NETs, thereby perpetuating a pathogenic cycle of NET release and IFN-I production.^11^

Anifrolumab is a fully human monoclonal antibody targeting the IFN-α receptor subunit 1, thereby blocking downstream IFN signalling.^12^ In phase IIb and phase III trials (A Phase II, Randomized Study to Evaluate the Efficacy and Safety of MEDI‐546 in Subjects with Systemic Lupus Erythematosus (MUSE); ClinicalTrials.gov identifier: NCT01438489, Treatment of Uncontrolled Lupus via the Interferon Pathway 1, Treatment of Uncontrolled Lupus via the Interferon Pathway 2 (TULIP-2)), anifrolumab demonstrated superiority to placebo in reducing global disease activity, cutaneous manifestations and corticosteroid requirements, with a favourable safety profile apart from a modest increase in herpes zoster infections.^13^,^15^ Following these results, anifrolumab became the second biologic approved for SLE after belimumab.^16 17^ However, its long-term role in real-world therapeutic algorithms remains to be established, as prospective observational data are limited. To address this gap, we established a longitudinal, multicentre observational cohort of patients with treatment-resistant SLE receiving anifrolumab in clinical practice (real-life longitudinal observational study to evaluate the efficacy and safety of the IFN-I receptor antagonist anifrolumab in patients with systemic lupus erythematosus). The objective was to evaluate clinical and laboratory responses as well as patient-reported outcomes over 12 months of follow-up.

## Methods

### Study design and participant selection

We performed a prospective, observational multicentre cohort study of patients with SLE treated with anifrolumab 300 mg intravenously every 4 weeks as part of routine clinical care of patients with SLE. Data collection was performed at two centres (Department of Internal Medicine 3 at FAU Erlangen-Nürnberg and Division of Rheumatology of the Nürnberg Hospital) between January 2024 and June 2025. This study was registered as a clinical study at the German Clinical Trials Register (DRKS00024360). Eligible patients were adults (aged ≥18 years) with a diagnosis of SLE according to the 2019 American College of Rheumatology/European League Against Rheumatism classification criteria, evidence of moderate-to-severe active disease at baseline and eligible for treatment with anifrolumab. Patients were excluded if they had active lupus nephritis at baseline, received anifrolumab outside the approved indication or were pregnant or breastfeeding. A total of 20 consecutive patients fulfilling these criteria were enrolled. The intention-to-treat (ITT) population comprised all 20 enrolled patients. Observed longitudinal analyses were based on patients with available follow-up data, while binary response end points were additionally analysed in the ITT population using non-responder imputation (ITT-NRI).

### Clinical, patient-reported and laboratory assessments

The primary end point was the change in the Systemic Lupus Erythematosus Disease Activity Index 2000 (SLEDAI-2K) total score from baseline to the end of study. Disease activity was measured by SLEDAI and its respective domain scores (vasculitic, renal, musculoskeletal, skin, immunological, constitutional and haematological). Response to therapy was determined by the achievement of a SLE Responder Index-4 (SRI-4), defined as ≥4-point reduction in SLEDAI. The proportion of patients reaching the Lupus Low Disease Activity State (LLDAS) and a clinical remission defined as achievement of the Definition of Remission in SLE (DORIS) criteria was also evaluated.

Joint involvement was assessed through tender and swollen joint counts (TJC, SJC) and the 28-joint Disease Activity Score based on C reactive protein (DAS28-CRP) score. Patient-reported and physician-reported disease activity were measured using global assessment scales, while patient-reported outcomes included pain, the Health Assessment Questionnaire Disability Index (HAQ-DI) and the Functional Assessment of Chronic Illness Therapy-Fatigue (FACIT-Fatigue) questionnaire.

Laboratory end points comprised haematological parameters (haemoglobin, leucocytes, neutrophils, lymphocytes, platelets), inflammatory markers (CRP, erythrocyte sedimentation rate (ESR)), autoantibodies (antidouble-stranded DNA (anti-dsDNA)) and complement components (C3 and C4).

### Assessment of NETs degradation products, chemokines, cytokines and low-density neutrophils

EDTA blood and serum samples were obtained from patients in the study cohort were obtained prior to treatment and at months 3, 6, 9 and 12. Serum samples were stored at −80°C until the final analyses, EDTA blood samples were processed within 5 hours after blood collection. Serum cytokine and chemokine concentrations were measured using the LegendPlex Assays (#740809 and #740003, BioLegend) according to manufacturer’s instructions. Serum was further analysed regarding neutrophil elastase (NE)-DNA and myeloperoxidase (MPO)-DNA complexes by ELISA. Briefly, plates were coated with 0.5 µg/mL of a human NE (#MAB91673, R&D) or MPO (#07-496-I, Merck KGaA) antibody and incubated for 18 hours at 4°C. Plates were washed with phosphate-buffered saline+0.05% Tween-20 (PBS-T) three times before blocking with 3% bovine serum albumin in PBS for 2 hours. Afterwards, plates were washed three times with PBS-T before adding 100 µL serum and incubating for 2 hours at room temperature with shaking. Subsequently, plates were washed three times with PBS-T before adding 100 µL DNA-peroxidase antibody 1:40 in incubation buffer (#11544675001, Merck KGaA). After 90 min incubation, plates were washed three times with PBS-T before adding 50 µL TMB substrate (#421101, BioLegend). The reaction was stopped after 60 min by adding 25% H_2_SO_4_ and measured at 450 nm with wavelength correction at 620 nm.

Anticoagulated blood was overlaid with Histopaque-1077 (#10771, Merck KGaA) and centrifuged at 1400 × g for 30 min without interruption. Polymorphonuclear and mononuclear (MN) density fractions were harvested separately and erythrocytes were lysed by osmotic lysis. Each fraction was stained with fluorescently-labelled antibodies (CD10-PE/Cy7, #982210, BioLegend; CD11b-VioGreen, #130-113-801, Milteny Biotec; CD15-APC, #301908, BioLegend; CD16-PacificBlue, #558122, BD Pharmingen; CD41a-AF700, #303728, BioLegend; CD49d-PE, #555503, BioLegend; CD66b-FITC, #IM0531U, Beckman Coulter; CD101-PerCP/Cy5.5, #331016, BioLegend) in FACS buffer (PBS+10% fetal bovine serum+5 mM EDTA) for 30 min at 4°C and acquired with the Gallios flow cytometer (Beckman Coulter). Analysis was performed with Kaluza Analysis Software (Beckman Coulter). Neutrophils were identified as SSc^high^/CD11b^+^/CD15^+^/CD16^+^/CD49d^−^ cells. Percentages of LDNs were calculated from flow cytometry data as the fraction of neutrophils in the MN density fraction. Merging linear mode data files from all time points allowed a bulk exploratory longitudinal phenotyping of LDN.

### Statistical analysis

Baseline continuous variables were summarised as mean±SD and categorical baseline variables, including LLDAS, were reported as counts and percentages. Longitudinal analyses were performed separately for each parameter using generalised additive models (GAMs). The models included time (months 0, 3, 6, 9, 12) as a smooth term and, when repeated measurements per subject were available, a patient-specific random intercept. Continuous outcomes were modelled with a Gaussian distribution, and binary outcomes with a binomial distribution and logit link. The smooth term used thin plate splines with a restricted number of knots to avoid overfitting, and models were estimated with restricted maximum likelihood. For binary response end points, including SRI-4, an ITT-NRI was additionally performed, in which patients who discontinued treatment were counted as non-responders at all subsequent time points. Estimated marginal means and 95% CIs were obtained for the prespecified months, based on predictions averaged across subjects and excluding random effects. Predictions for binary outcomes were back-transformed from the logit scale. The overall time effect was assessed using the approximate F-test for the smooth term. Results were summarised in tables and plots with error bars. Analyses were performed in R V.4.5.1 using the mgcv package for modelling.^18^

## Results

### Baseline demographic and clinical characteristics

Twenty patients were enrolled in the study (ITT population), 85% of whom were female. The mean age at baseline was 44.8±11.2 years and the mean disease duration was 11±7.7 years (table 1). Seventeen patients completed follow-up and were included in the observed longitudinal analyses. The most frequent SLE manifestations throughout patients’ medical history were musculoskeletal (95%), immunological (95%) and cutaneous (90%), followed by haematological (55%), renal manifestations without nephritis (40%) and vasculitis (25%). Serositis (15%) and constitutional manifestations (10%) were uncommon. No patient had a history of central nervous system (CNS) involvement.

**Table 1:** Baseline demographic and clinical characteristics

	N=20
Demographics	
Female sex, n (%)	17 (85%)
Age (years), mean (SD)	44.8 (11.2)
Disease duration (years), mean (SD)	11 (7.7)
Clinical manifestations, n (%)	
CNS	0 (0%)
Vasculitis	5 (25%)
Renal	8 (40%)
Musculoskeletal	19 (95%)
Skin	18 (90%)
Serositis	3 (15%)
Haematological	11 (55%)
Constitutional	2 (10%)
Immunological	19 (95%)
Therapy	
Glucocorticoids	
Previous use, n (%)	7 (35%)
Current use, n (%)	13 (65%)
Dose (mg/day), mean (SD)	10.1 (8.0)
Concurrent csDMARD, n (%)	
Hydroxychloroquine	18 (90%)
Methotrexate	3 (15%)
Azathioprine	2 (10%)
Mycophenolate	7 (35%)
Previous therapies, n (%)	
Hydroxychloroquine	20 (100%)
Methotrexate	11 (55%)
Azathioprine	9 (45%)
Mycophenolate	15 (75%)
Cyclophosphamide	1 (5%)
Leflunomide	1 (5%)
Belimumab	11 (55%)
Rituximab	5 (25%)

CNS, central nervous system; csDMARD, conventional synthetic disease-modifying antirheumatic drug.

Eleven patients (55%) had previously failed belimumab therapy and five of them (25%) remained refractory despite subsequent B-cell depletion with rituximab. At baseline, most patients were receiving concomitant therapy with hydroxychloroquine (18/20, 90%), followed by mycophenolate mofetil (7/20, 35%), methotrexate (3/20, 15%) and azathioprine (2/20, 10%). Seven patients were not receiving glucocorticoids at baseline. The remaining 13 were receiving a mean daily prednisolone-equivalent dose of 10.1±8.0 mg.

Baseline disease activity was moderate, with a mean SLEDAI total score of 10.9±5.0. At baseline, the most prominent domain contributions were musculoskeletal (3.06±1.75), immunological (2.59±1.54) and skin (2.47±1.33). Vasculitic manifestations were less common (1.88±3.50), while renal (0.71±1.57) and haematological (0.24±0.44) activity was less frequent. None of the patients were in low disease activity at baseline (LLDAS: 0%). Musculoskeletal involvement was reflected in a mean TJC of 4.4±8.0 and SJC of 1.47±2.50. The mean DAS28-CRP was 3.19±1.48 units. The mean reported pain score was 4.00±2.70 cm and mean patient global activity was 4.58±2.19 cm. The mean Physician Global Assessment (PGA) at baseline was 1.65±0.46 cm. Functional impairment and fatigue were present, with a HAQ-DI of 1.27±0.73 and a FACIT-Fatigue score of 23±14. Inflammatory markers were increased at baseline (CRP 12±19 mg/L; ESR 13.7±7.1 mm/hour). Complement levels were at the lower limit of normal (C3 90±33 mg/dL; C4 15±8 mg/dL) and anti-dsDNA antibody titres were markedly elevated (192±188 IU/mL).

### Disease activity and clinical outcomes

**Figure 1:**
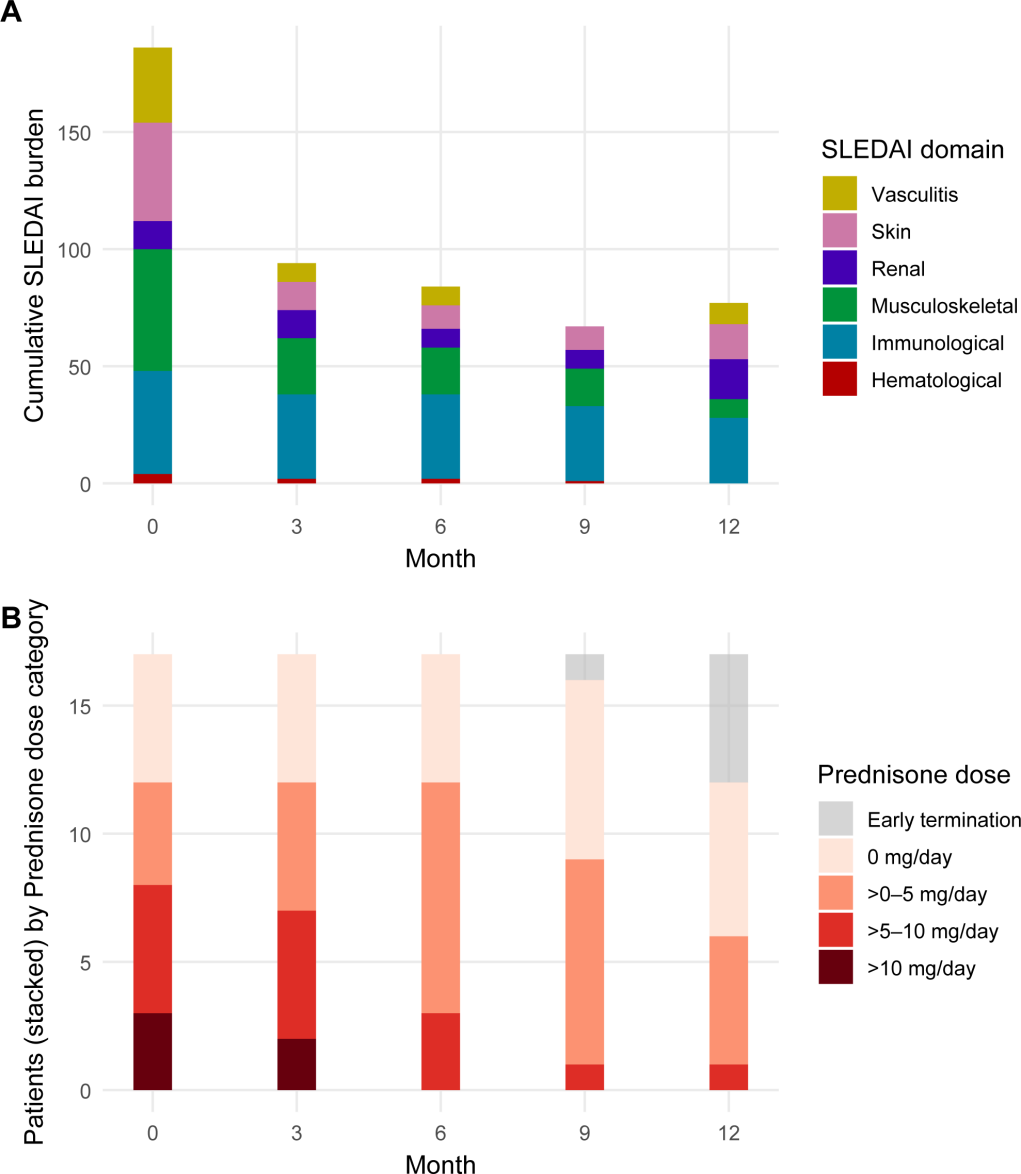
Longitudinal changes in disease activity and glucocorticoid use over 12 months. (**A**) Cumulative Systemic Lupus Erythematosus Disease Activity Index (SLEDAI) burden across all patients at each visit, decomposed by individual SLEDAI domains. Stacked bars represent the summed contribution of each domain to overall disease activity at each time point. (**B**) Distribution of patients across prednisone dose categories over time. Stacked bars show the number of patients within each dose category at each visit, including early termination.

Swimmer plots and GAMs demonstrated substantial improvements in overall and domain-specific disease activity across 12 months (table 2, online supplemental table 1, figures1A  2). The SLEDAI total score decreased from 11 (95% CI 9 to 13) at baseline to 3.1 (95% CI 1.1 to 5.1) at month 12 (p<0.001). Improvements were most pronounced in the musculoskeletal (p<0.001), skin (p<0.001) and immunological (p=0.015) domains, whereas haematological and renal components did not change substantially; vasculitic activity was infrequent and showed only limited changes over time. Constitutional activity remained absent throughout the observation period. Using ITT-NRI, SRI-4 response rates were 64.7% at month 3, 76.5% at month 6, 70.6% at month 9 and 64.7% at month 12. In contrast, model-based estimates from longitudinal analyses suggested a probability of 0.97 (95% CI 0.3 to 1) at month 12 (p=0.009). By month 12, 86% of patients attained LLDAS. Model-based estimates showed a corresponding increase from 0 at baseline to 0.86 (95% CI 0.5 to 1.0) at month 12 (p<0.001). The proportion of patients who achieved DORIS remission criteria increased from zero at 3 months to 15% (n=3) at 6 months, 45% (n=9) at 9 months and finally to 50% (n=10) at 12 months. Model-based estimates suggested a probability of DORIS remission of 0.89 (95% CI 0.6 to 1.0) at month 12 (p<0.001).

**Table 2:** Baseline clinical characteristics and longitudinal changes in clinical, laboratory and patient-reported outcomes

Outcome	Baseline	Month 3	Month 6	Month 9	Month 12
Clinical parameters					
SLEDAI: CNS	0 (0 to 0)	0 (0 to 0)	0 (0 to 0)	0 (0 to 0)	0 (0 to 0)
SLEDAI: vasculitis	1.62 (0.5 to 2.7)	0.83 (0 to 1.7)	0.34 (0 to 1.3)	0.21 (0 to 1.1)*	0.3 (0 to 1.5)*
SLEDAI: renal	0.68 (0 to 1.5)	0.61 (0 to 1.3)	0.58 (0 to 1.4)	0.68 (0 to 1.5)	0.91 (0 to 1.9)
SLEDAI: musculoskeletal	2.9 (2 to 3.8)	1.72 (0.8 to 2.6)*	1.2 (0.4 to 2)***	1 (0.2 to 1.9)***	0.62 (0 to 1.5)***
SLEDAI: skin	2.32 (1.6 to 3)	0.98 (0.4 to 1.6)**	0.48 (0 to 1)***	0.7 (0.1 to 1.3)***	1.11 (0.4 to 1.8)*
SLEDAI: serositis	0 (0 to 0)	0 (0 to 0)	0 (0 to 0)	0 (0 to 0)	0 (0 to 0)
SLEDAI: haematological	0.17 (0 to 1)	0 (0 to 1)	0 (0 to 1)	0 (0 to 1)	0 (0 to 1)
SLEDAI: constitutional	0 (0 to 0)	0 (0 to 0)	0 (0 to 0)	0 (0 to 0)	0 (0 to 0)
SLEDAI: immunological	2.46 (1.8 to 3.1)	2.29 (1.6 to 3)*	2.13 (1.5 to 2.8)*	1.97 (1.3 to 2.6)*	1.8 (1.1 to 2.5)*
SLEDAI: total score	11 (9 to 13)	6.07 (4.2 to 7.9)***	4.52 (2.8 to 6.3)***	4.38 (2.5 to 6.2)***	3.1 (1.1 to 5.1)***
SLEDAI Response (SRI-4)	0 (0 to 0.5)	0.64 (0.1 to 1)*	0.92 (0.3 to 1)*	0.89 (0.2 to 1)**	0.97 (0.3 to 1)**
LLDAS	0 (0 to 0.2)	0.08 (0 to 0.3)***	0.27 (0.1 to 0.6)***	0.6 (0.3 to 0.9)***	0.86 (0.5 to 1)***
DORIS (remission)	0 (0 to 0.1)	0.02 (0 to 0.2)***	0.14 (0 to 0.4)***	0.53 (0.3 to 0.8)***	0.89 (0.6 to 1)***
Physician Global Assessment	1.57 (1.3 to 1.9)	1.19 (0.9 to 1.5)***	0.83 (0.6 to 1.1)***	0.49 (0.2 to 0.7)***	0.16 (0 to 0.5)***
SJC	1.37 (0.1 to 2.7)	1.29 (0 to 2.6)	1.22 (0 to 2.5)	1.14 (0 to 2.4)	1.06 (0 to 2.4)
TJC	3.92 (1.6 to 6.2)	2.36 (0.3 to 4.4)	1.54 (0 to 3.5)	1.24 (0 to 3.2)*	0.95 (0 to 3.2)**
DAS28-CRP	3.12 (2.5 to 3.8)	2.93 (2.3 to 3.6)***	2.75 (2.1 to 3.4)***	2.57 (1.9 to 3.2)***	2.39 (1.7 to 3)***
Patient-reported outcomes					
Pain (patient-rated)	3.98 (2.6 to 5.4)	3.76 (2.4 to 5.1)*	3.54 (2.2 to 4.9)*	3.32 (1.9 to 4.7)*	3.1 (1.7 to 4.5)*
Global activity (patient-rated)	4.33 (2.9 to 5.7)	3.83 (2.5 to 5.2)	3.5 (2.2 to 4.8)	3.31 (2 to 4.6)*	3.18 (1.7 to 4.6)*
HAQ-DI	1.24 (0.8 to 1.7)	1.09 (0.7 to 1.5)	1 (0.6 to 1.4)	1 (0.6 to 1.4)	1.08 (0.6 to 1.5)
FACIT-Fatigue	24 (15 to 33)	25 (16 to 34)	25 (16 to 34)	24 (16 to 33)	23 (14 to 32)
Laboratory parameters					
Haemoglobin (g/L)	124 (116 to 131)	125 (118 to 132)**	126 (119 to 133)**	127 (120 to 135)**	129 (121 to 136)**
Leucocytes (10^3^/µL)	6.05 (4.6 to 7.5)	6.39 (5 to 7.8)**	6.74 (5.3 to 8.1)**	7.08 (5.7 to 8.5)**	7.43 (5.9 to 8.9)**
Neutrophils (%)	63 (58 to 67)	64 (60 to 68)	65 (61 to 69)	66 (62 to 70)	67 (63 to 72)
Lymphocytes (%)	24 (20 to 28)	23 (20 to 26)	23 (20 to 26)	22 (20 to 25)	22 (18 to 26)
Platelets (10^3^/µL)	268 (213 to 323)	278 (224 to 332)**	289 (235 to 342)**	299 (245 to 353)**	309 (254 to 365)**
CRP (mg/L)	12 (8 to 15)	11 (8 to 14)	9.82 (7.3 to 12.3)	8.91 (6 to 11.8)	8 (4.1 to 11.9)
ESR (mm/hour)	14 (7 to 22)	15 (9 to 22)	16 (11 to 22)	18 (11 to 24)	19 (11 to 26)
ds-DNA (IU/mL)	240 (72 to 407)	225 (70 to 380)	211 (59 to 362)	196 (38 to 353)	181 (9 to 354)
Complement C3 (mg/dL)	90 (73 to 107)	91 (74 to 108)	91 (75 to 108)	92 (75 to 109)	93 (76 to 110)
Complement C4 (mg/dL)	16 (12 to 19)	15 (11 to 19)***	15 (11 to 18)***	14 (10 to 18)***	13 (10 to 17)***

Values are estimated marginal means with 95% CIs from generalised additive models. P values reflect model-based contrasts between baseline and follow-up visits at months 3, 6, 9 and 12. Significance indicators: *p<0.05; **p<0.01; ***p<0.001. Exact p values are provided in the online supplemental material.

CNS, central nervous system; CRP, C reactive protein; DAS28-CRP, 28-joint Disease Activity Score based on C reactive protein; DORIS, Definition of Remission in SLE; ds-DNA, double-stranded DNA; ESR, erythrocyte sedimentation rate; FACIT-Fatigue, Functional Assessment of Chronic Illness Therapy-Fatigue; HAQ-DI, Health Assessment Questionnaire Disability Index; LLDAS, Lupus Low Disease Activity State; SJC, swollen joint count; SLEDAI, Systemic Lupus Erythematosus Disease Activity Index; SRI-4, SLE Responder Index-4; TJC, tender joint count.

**Figure 2:**
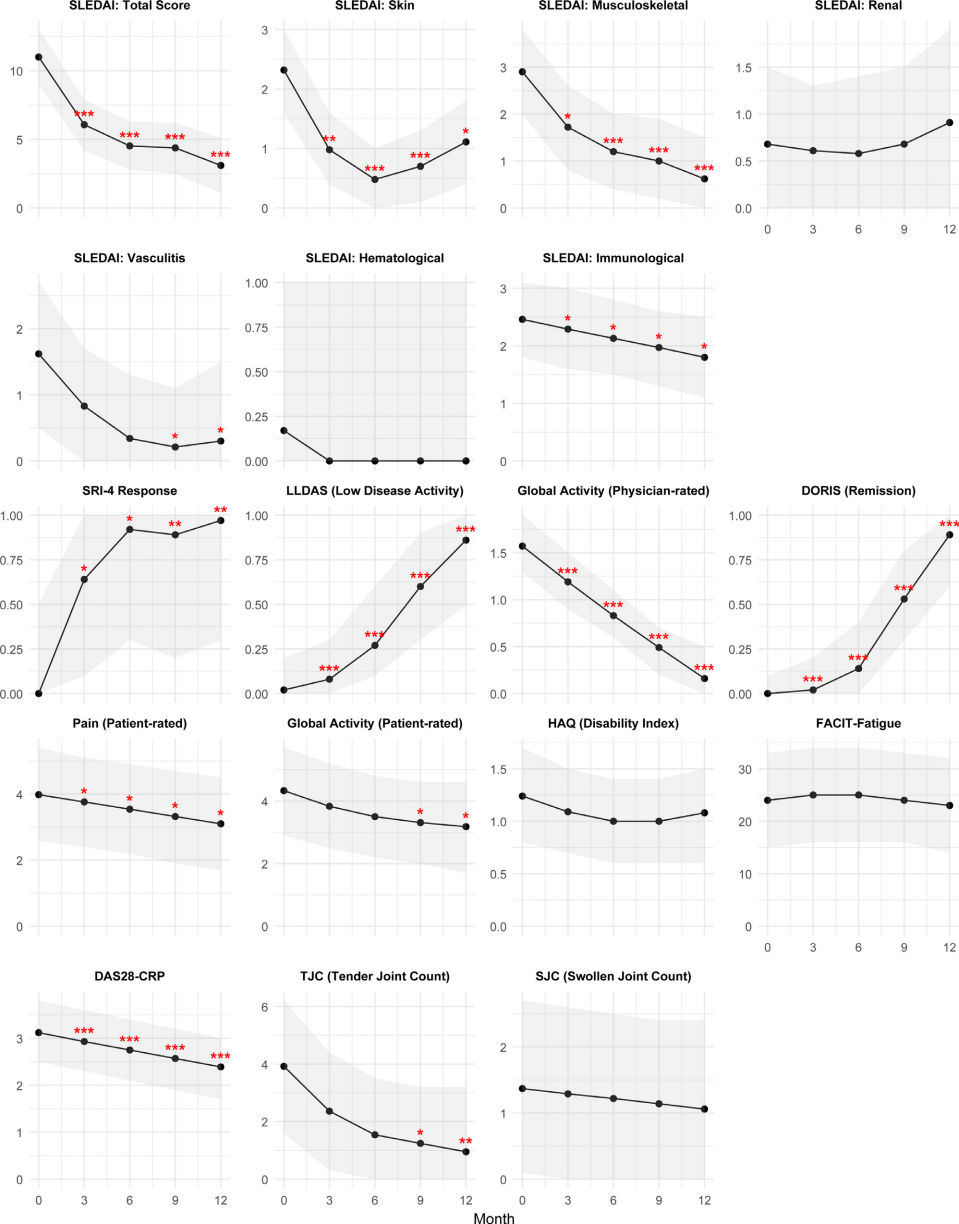
Longitudinal changes in clinical disease activity and patient-reported outcomes. Estimated marginal means with 95% CIs derived from generalised additive models for clinical disease activity measures, physician-reported and patient-reported outcomes and response indices assessed at baseline and months 3, 6, 9 and 12. Symbols indicate the strength of the overall time effect. DAS28, disease activity score 28; FACIT-Fatigue, Functional Assessment of Chronic Illness Therapy-Fatigue; HAQ, Health Assessment Questionnaire; LLDAS, Lupus Low Disease Activity State; SJC, swollen joint count; SLEDAI, Systemic Lupus Erythematosus Disease Activity Index; SRI-4, Systemic Lupus Erythematosus Responder Index-4; TJC, tender joint count.

Joint involvement also improved. DAS28-CRP declined steadily from 3.12 (95% CI 2.5 to 3.8) to 2.39 (95% CI 1.7 to 3) (p<0.001). TJCs decreased significantly (p=0.009), while SJCs did not change significantly (p=0.261). PGA improved substantially, falling from 1.57 (95% CI 1.3 to 1.9) to 0.16 (95% CI 0 to 0.5) (p<0.001). Patient-reported outcomes showed modest but significant improvements in pain (p=0.042) and global activity (p=0.014), whereas fatigue, as measured by FACIT-Fatigue, remained unchanged (p=0.524).

There were no meaningful differences in clinical response outcomes between patients with prior exposure to B-cell-targeted therapies and those who were naïve to B-cell-targeted therapy.

### Glucocorticoid use and concomitant therapies

We observed a reduction in glucocorticoid use in our cohort during treatment with anifrolumab. The mean daily dose was significantly reduced from 10.1 to 5.3±5.2 mg/day (p=0.036). At baseline, 13 patients were receiving glucocorticoids. By the end of follow-up, 11/13 patients were able to taper or maintain their daily prednisolone-equivalent dose at ≤5 mg/day, corresponding to an increase from 2/20 (10%) to 11/20 (55%) in the overall cohort. Three patients were able to discontinue glucocorticoid therapy completely. Regarding the two patients who continued receiving >5 mg/day of glucocorticoids, in one case prednisolone was tapered from 15 to 10 mg/day; however, further dose reductions resulted in worsening myalgia and stiffness. In the second case, anifrolumab was discontinued after <3 months of treatment due to adverse events (AEs) (figure 1B).

In six cases, the dose of the concomitant csDMARD was also reduced. MMF was tapered to a lower dose in two patients and discontinued in three patients. In one additional patient, the weekly dose of methotrexate was reduced.

### Safety and drug persistence

Drug persistence was 65.0% at 12 months by Kaplan-Meier analysis (95% CI 0.40 to 0.82). In three cases, anifrolumab was discontinued before month 3 due to AEs (muscle pain and bronchitis, n=1; flare of SLE with nephritis and COVID-19, n=1; headache, joint pain and dyspnoea, n=1). Seventeen patients continued regular follow-up and were included in the final analysis (table 2, online supplemental table 1). Of them, four patients did not continue anifrolumab therapy after 9 months due to ongoing remission (n=2), lack of response with respect to arthritis (n=1) and death due to heart failure and influenza A virus infection complicated by bacterial superinfection and sepsis (n=1). The latter case involved a female patient aged 56 years, current smoker with comorbid congestive heart failure and ischaemic heart disease, not vaccinated for influenza, who was receiving co-therapy with hydroxychloroquine and mycophenolate, but no oral glucocorticoids.

Throughout follow-up, a total of 55 AEs were recorded, including four serious AEs (two cases of pneumonia requiring hospital admission, one in situ tongue carcinoma and one death due to influenza A virus infection and sepsis). Infectious AEs were the most prevalent (n=33, 60%) and were mostly mild and self-resolving, comprising airway infections including COVID-19 (n=23), urinary tract infections (n=3), skin infections (n=3), oral candidiasis (n=1), varicella zoster virus reactivation (n=1) and other mild infections (n=2). Non-infectious AEs included gastrointestinal symptoms (n=5), headache (n=5), myalgia (n=4), hair loss (n=2), SLE flares (n=2), erythema (n=1) and other mild AEs (n=3).

### Laboratory biomarkers

**Figure 3:**
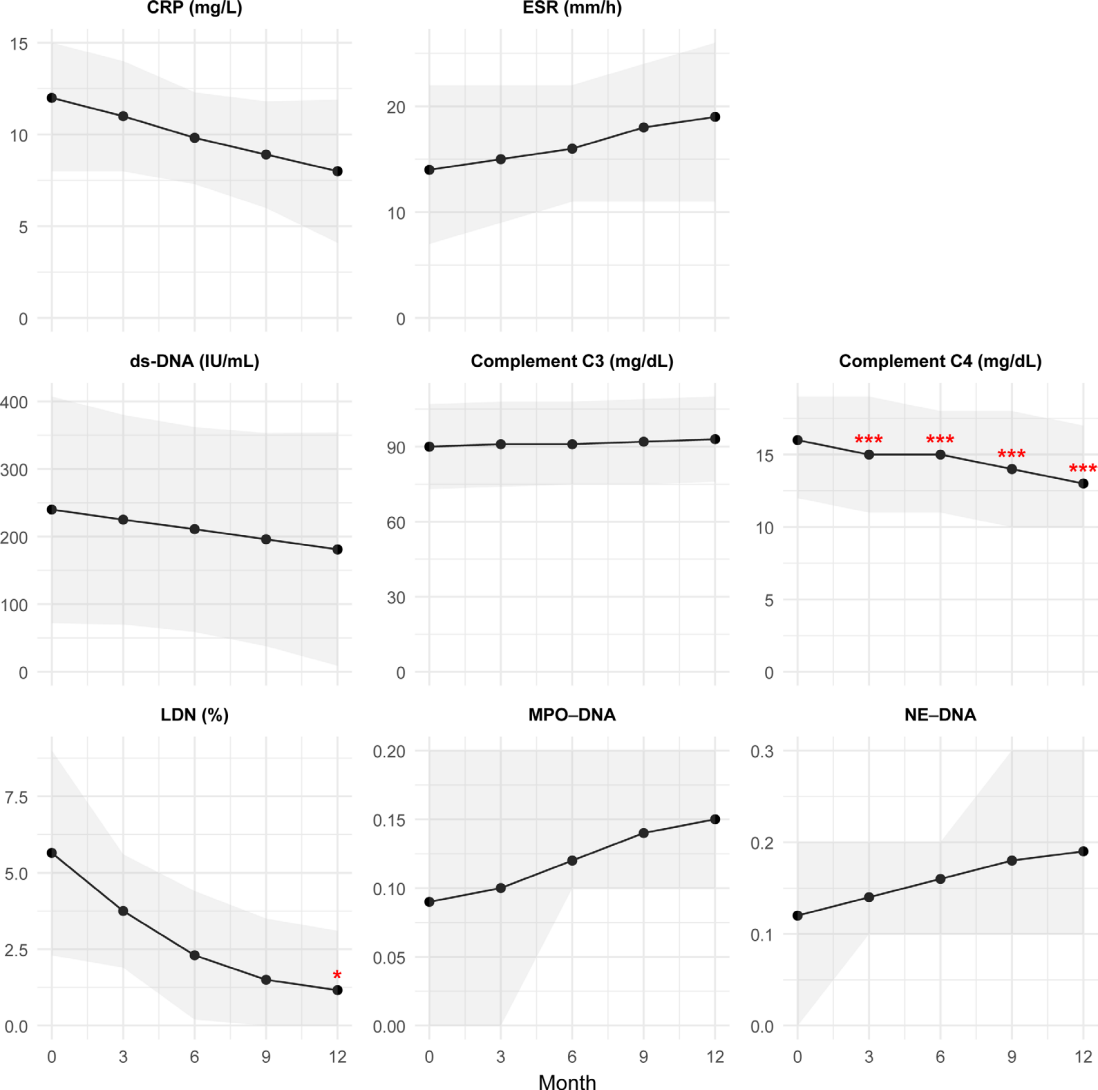
Laboratory markers, autoantibodies and NET-related parameters over time. Longitudinal trajectories of inflammatory markers, serological parameters, complement levels, LDNs and neutrophil extracellular trap-associated markers over 12 months. Points represent estimated marginal means with shaded 95% CIs. Significant overall time effects are indicated within panels. CRP, C reactive protein; ESR, erythrocyte sedimentation rate; LDN, low-density neutrophils; MPO, myeloperoxidase; NE, neutrophil elastase.

Haematological recovery was evident, with significant increases in haemoglobin (p=0.008), leucocyte counts (p=0.004) and platelet counts (p=0.002) during follow-up (table 2, online supplemental table 1, figure 3). Neutrophil and lymphocyte counts did not change. Inflammatory markers, including CRP (p=0.222) and ESR (p=0.387), remained stable over time. Complement C4 declined significantly (p<0.001), but always stayed within the normal range, while complement C3 (p=0.217) and anti-dsDNA antibody titres (p=0.457) did not show significant changes.

### Cytokines and NETs degradation products

Longitudinal modelling showed a substantial decrease in the percentage of LDNs from 5.65% to 1.16% (p=0.037). NETs degradation products did not significantly change (table 3, online supplemental table 2, figure 3). Exploratory longitudinal phenotyping of LDNs additionally suggested temporal changes in CD66b and CD41a expression intensity during follow-up, consistent with phenotypic modulation of this subset under treatment (online supplemental figure 1).

**Table 3:** Longitudinal changes in cytokines and neutrophil extracellular trap parameters

Outcome	Baseline	Month 3	Month 6	Month 9	Month 12
LDNs and NETs					
LDN (%)	5.65 (2.3 to 9)	3.75 (1.9 to 5.6)	2.3 (0.2 to 4.4)	1.5 (0 to 3.5)	1.16 (0 to 3.1)*
MPO-DNA	0.09 (0 to 0.2)	0.1 (0 to 0.2)	0.12 (0.1 to 0.2)	0.14 (0.1 to 0.2)	0.15 (0.1 to 0.2)
NE-DNA	0.12 (0 to 0.2)	0.14 (0.1 to 0.2)	0.16 (0.1 to 0.2)	0.18 (0.1 to 0.3)	0.19 (0.1 to 0.3)
Neutrophil chemokines					
RANTES	26 (21 to 30)	24 (20 to 28)***	22 (18 to 26)***	20 (16 to 25)***	19 (14 to 23)***
MCP-1	1218 (536 to 1900)	1136 (468 to 1803)*	1054 (394 to 1713)*	972 (313 to 1630)*	889 (224 to 1554)*
TARC	3118 (2241 to 3996)	3045 (2317 to 3773)	2972 (2337 to 3607)	2898 (2288 to 3508)	2824 (2169 to 3480)
Eotaxin	167 (85 to 249)	154 (75 to 234)*	141 (63 to 219)*	129 (51 to 206)*	116 (37 to 195)*
IP-10	4.91 (3.8 to 6)	4.64 (3.6 to 5.7)***	4.38 (3.4 to 5.4)***	4.11 (3.1 to 5.1)***	3.85 (2.8 to 4.9)***
IL-8	20 (16 to 25)	19 (14 to 23)***	17 (13 to 21)***	15 (11 to 19)***	13 (9 to 17)***
MIP-1β	12 (10 to 14)	11 (10 to 13)***	11 (9 to 12)***	9.71 (8.1 to 11.3)***	8.89 (7.3 to 10.5)***
I-TAC	11 (9 to 14)	11 (8 to 13)***	9.74 (7.6 to 11.8)***	8.98 (6.9 to 11.1)***	8.22 (6.1 to 10.4)***
GRO-α	3862 (0 to 13343)	4163 (0 to 12840)	4464 (0 to 12712)	4762 (0 to 12966)	5057 (0 to 13565)
MIP-3α	8.12 (6.8 to 9.4)	7.47 (6.4 to 8.5)*	6.84 (5.8 to 7.9)*	6.26 (5.1 to 7.4)**	5.75 (4.6 to 6.9)***
ENA-78	4.35 (3.3 to 5.3)	4.12 (3.1 to 5.1)***	3.9 (2.9 to 4.9)***	3.67 (2.7 to 4.6)***	3.45 (2.5 to 4.4)***
Cytokines					
IL-1β	115 (108 to 123)	115 (108 to 122)	115 (108 to 122)	115 (108 to 122)	115 (108 to 122)
IL-6	85 (78 to 92)	84 (78 to 91)	83 (77 to 89)	83 (77 to 89)	82 (76 to 88)
TNF-α	229 (187 to 272)	210 (179 to 240)	191 (158 to 223)	175 (139 to 210)*	162 (128 to 197)*
IFN-α2	48 (44 to 53)	49 (45 to 53)	49 (45 to 53)	49 (45 to 53)	49 (45 to 53)
IFN-γ	147 (120 to 174)	147 (123 to 171)	146 (123 to 168)	145 (123 to 168)	145 (121 to 169)
IL-12p70	53 (50 to 56)	53 (50 to 55)	52 (50 to 55)	52 (49 to 55)	52 (49 to 54)
IL-17A	16 (14 to 17)	16 (15 to 17)	16 (15 to 16)	16 (15 to 16)	16 (15 to 17)
IL-18	522 (456 to 587)	523 (463 to 583)	524 (467 to 581)	525 (468 to 582)	526 (467 to 586)
IL-23	142 (131 to 152)	143 (133 to 153)	145 (135 to 154)	146 (137 to 156)	148 (138 to 157)
IL-10	38 (32 to 43)	39 (35 to 42)	39 (35 to 44)	39 (34 to 43)	37 (33 to 41)
IL-33	587 (547 to 626)	588 (551 to 625)	590 (554 to 626)	591 (555 to 627)	593 (556 to 630)

Values are estimated marginal means with 95% CIs from generalised additive models. P values reflect model-based contrasts between baseline and follow-up visits at months 3, 6, 9 and 12. Outcomes include circulating cytokines, chemokines and NET-associated markers measured longitudinally to characterise immunological changes over the 12-month follow-up. Significance indicators: *p<0.05; **p<0.01; ***p<0.001. Exact p values are provided in the online supplemental material.

ENA-78, epithelial neutrophil-activating peptide 78 (CXCL5); GRO-α, growth-related oncogene alpha (CXCL1); IFN, interferon; IL, interleukin; IP-10, interferon gamma-induced protein 10 (CXCL10); I-TAC, interferon-inducible T-cell alpha chemoattractant (CXCL11); LDN, low-density neutrophil; MCP-1, monocyte chemoattractant protein 1 (CCL2); MIP-3α, macrophage inflammatory protein 3 alpha (CCL20); MIP-1β, macrophage inflammatory protein 1 beta (CCL4); MPO-DNA, myeloperoxidase-DNA complexes; NE-DNA, neutrophil elastase-DNA complexes; RANTES, regulated on activation normal T cell expressed and secreted (CCL5); TARC, thymus-regulated and activation-regulated chemokine (CCL17); TNF, tumour necrosis factor.

**Figure 4:**
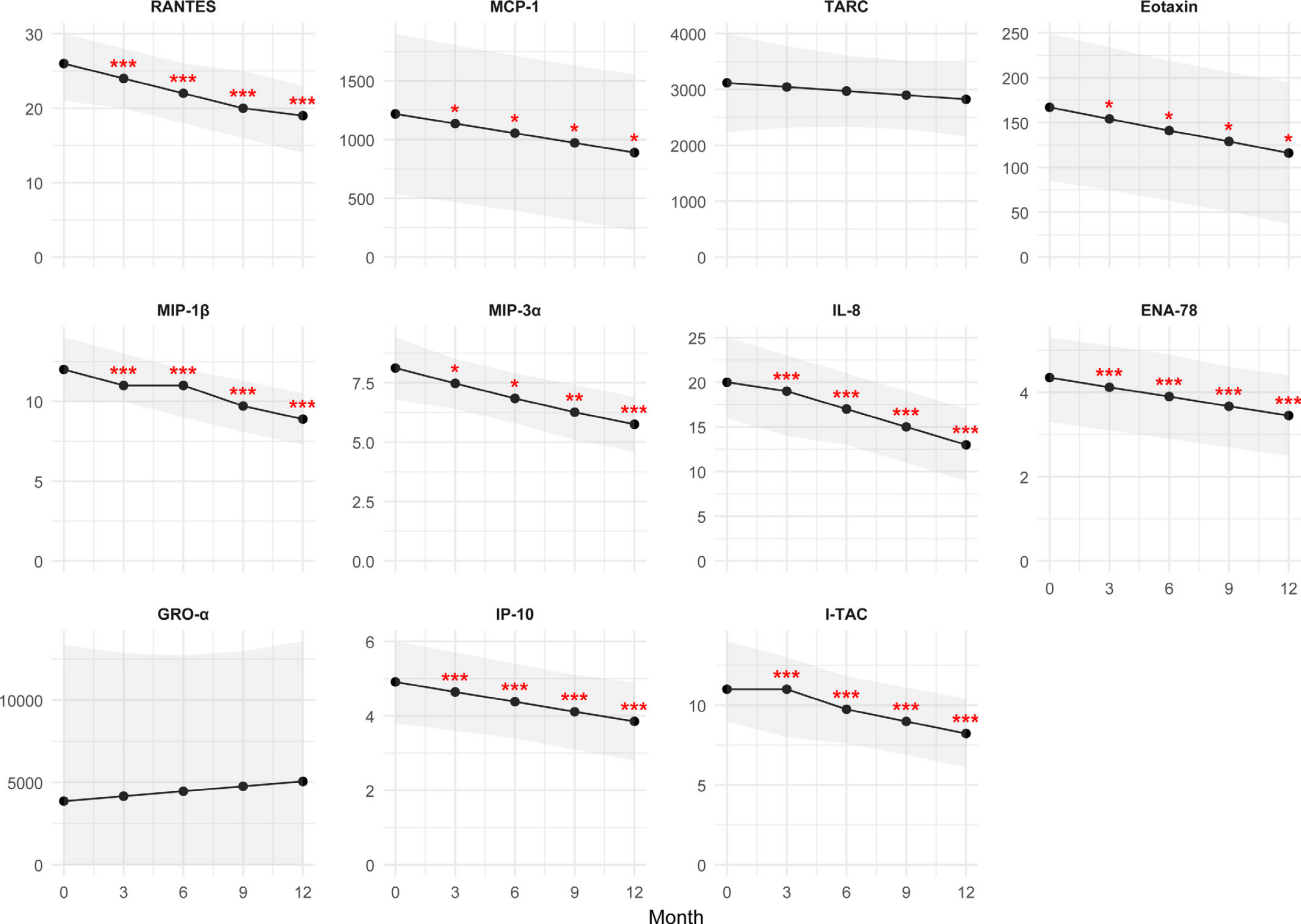
Chemokine profile dynamics during follow-up. Changes in serum chemokine concentrations over 12 months shown as estimated marginal means with 95% CIs. Several IFN-related and pro-inflammatory chemokines show consistent downward trends during follow-up. Asterisks indicate the magnitude of the overall time effect. ENA-78, epithelial-derived neutrophil-activating protein-78 (CXCL5); GRO-α, growth-regulated oncogene-α (CXCL1); IL, interleukin; IP-10, interferon-γ-induced protein-10 (CXCL10); I-TAC, interferon-inducible T-cell α chemoattractant (CXCL11); MCP-1, monocyte chemoattractant protein-1 (CCL2); MIP-1β and MIP-3α, macrophage inflammatory protein-1β (CCL4) and MIP-3α (CCL20); RANTES, regulated on activation, normal T-cell expressed and secreted (CCL5); TARC, thymus-regulated and activation-regulated chemokine (CCL17).

Several neutrophil-related chemokines significantly declined. Hence, regulated on activation normal T cell expressed and secreted (p<0.001), monocyte chemoattractant protein 1 (p=0.018), eotaxin (p=0.010) and interferon gamma-induced protein 10 (IP-10) (p<0.001) all showed steady reductions. Interleukin (IL)-8, macrophage inflammatory protein 1 beta, interferon-inducible T-cell alpha chemoattractant (I-TAC), macrophage inflammatory protein 3 alpha and epithelial-derived neutrophil-activating protein-78 also decreased significantly (all p<0.001). Growth-regulated oncogene-α remained unchanged (p=0.749) and thymus-regulated and activation-regulated chemokine, and IL-6 showed no meaningful changes (table 3, online supplemental table 2, figure 4).

**Figure 5:**
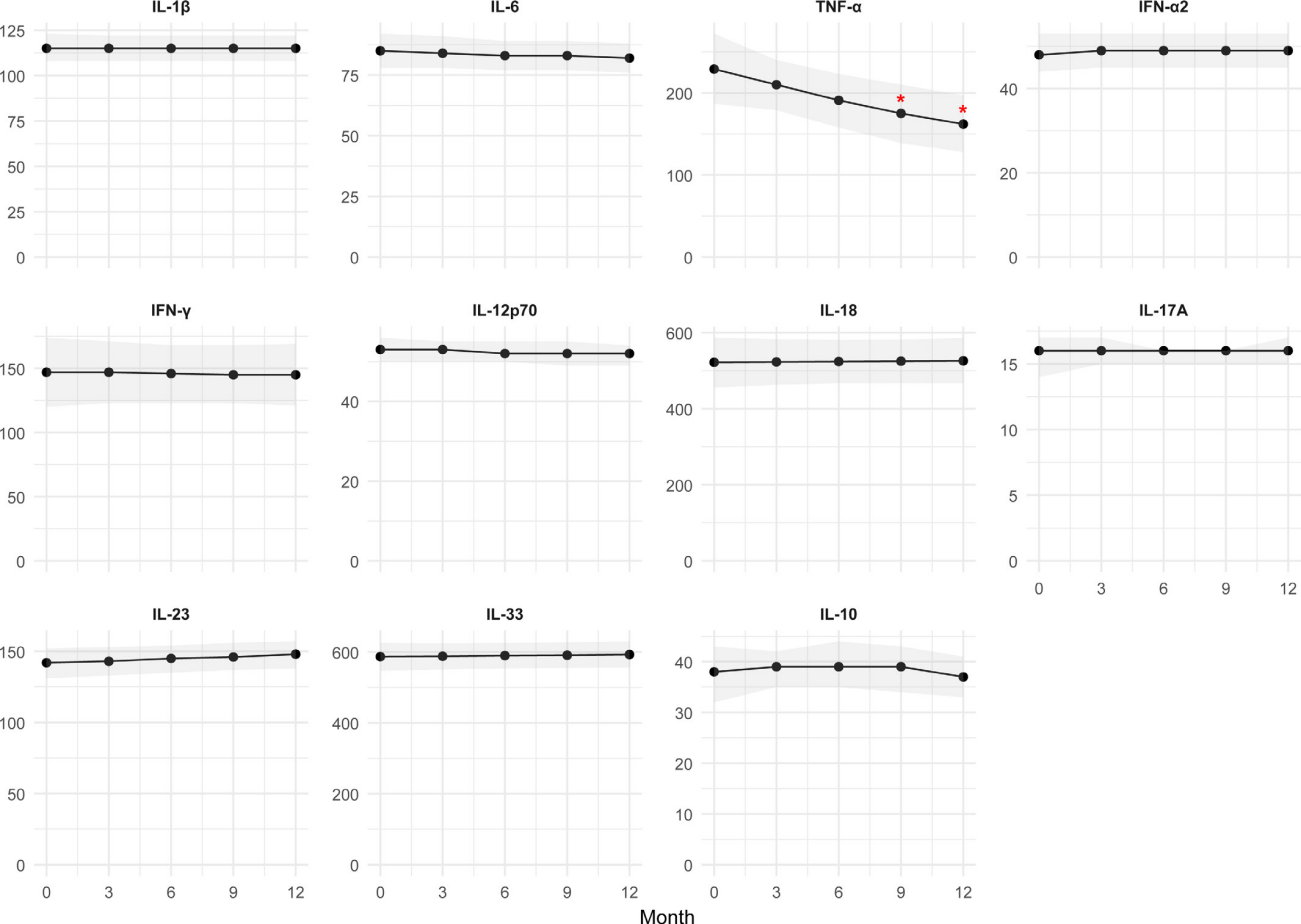
Cytokine concentrations during 12 months of treatment. Longitudinal analysis of serum cytokines across innate, adaptive and IFN-related pathways presented as estimated marginal means with 95% CIs from generalised additive models. Most cytokines remained stable, with selective changes observed for individual mediators over time. IFN, interferon; IL, interleukin; TNF, tumour necrosis factor.

Most cytokines linked to IFN or T-cell pathways remained stable, including IFN-α2, IFN-γ, IL-12p70, IL-17A, IL-18, IL-23, IL-33 and IL-10. TNF-α decreased significantly (p=0.014). Overall, cytokine and chemokine profiles showed broad decreases in inflammatory mediators over 12 months, while NETs degradation products remained largely unchanged (table 3, online supplemental table 2, figure 5).

## Discussion

Our real-world observations indicate that anifrolumab is safe and effective for sustained treatment of moderate-to-severe SLE without active neurological involvement. Over 12 months, we observed a substantial reduction in global disease activity, with the mean SLEDAI-2K score decreasing by roughly 7 points. By study end, SRI-4 response rates reached 64.7% in ITT-NRI, whereas model-based longitudinal estimates suggested a high probability of response. In parallel, 86% attained LLDAS and 50% achieved DORIS remission state. Achieving LLDAS and DORIS remission are key therapeutic goals in SLE and are associated with lower mortality, reduced organ damage, fewer flares and improved quality of life.^19 20^ Notably, anifrolumab therapy enabled tapering or discontinuation of glucocorticoids in almost all patients receiving daily prednisolone, suggesting that these improvements likely reflect a treatment effect rather than spontaneous remission. Supporting this claim, concomitant csDMARDs could be tapered in six cases.

Our findings align with pivotal trials. In TULIP-2, 47.8% of patients receiving anifrolumab achieved a British Isles Lupus Collaborating Group-based Composite Lupus Assessment response vs 31.5% on placebo.^15^ In the phase IIb MUSE study, anifrolumab reduced disease activity across multiple end points.^13^ The magnitude of SLEDAI reduction in our cohort (from ~11 to ~3) exceeds trial averages. Improvements were more marked in the musculoskeletal, skin, immunological and haematological domains, consistent with trial analyses.^21^ Musculoskeletal manifestations improved steadily, with reductions in DAS28-CRP and TJCs. SJCs changed little, possibly due to low baseline activity or the non-erosive nature of lupus arthritis, mirroring observations from TULIP-2.^21^

Skin manifestations showed one of the most robust responses. Patients with active rashes improved substantially, in line with trial data showing ≥50% Cutaneous Lupus Erythematosus Disease Area and Severity Index (CLASI) reduction in nearly half of anifrolumab-treated patients by week 12,^21^ as well as reports of dramatic responses in refractory cutaneous lupus.^22^ Of note, the recurrence of mild erythema following glucocorticoid tapering in two patients led to a transient increase in skin SLEDAI scores in two patients. Although CLASI was not systematically assessed, cutaneous involvement in both patients remained substantially milder than at baseline. These findings reinforce the importance of IFN blockade in cutaneous SLE. Renal and neuropsychiatric efficacy could not be assessed, as patients with active lupus nephritis or CNS involvement were excluded.

Clinical improvements were not paralleled by improvement in serological activity. Anti-dsDNA titres remained elevated, C3 levels were stable and C4 even decreased slightly, although remaining within the normal range, contrasting the serological effects seen with B-cell-targeted therapies.^21^ This likely reflects anifrolumab’s mechanism, which modulates IFN-driven inflammation without directly targeting B cells or plasma cells. This pattern resembles the ‘serologically active, clinically quiescent’ phenotype in SLE.^23^ Long-term observation of larger cohorts will be needed to confirm this phenomenon and characterise whether it could be associated with a higher risk of flares.

Patient-reported outcomes improved partially. Global disease activity and pain decreased, consistent with phase III trial data.^24^ Fatigue, however, did not improve. In SLE, fatigue is multifactorial and may persist despite control of immunological activity. Although IFN-I signalling has been linked to fatigue, anifrolumab trials show only modest effects on fatigue.^24^ Concomitant fibromyalgia, chronic pain, sleep disturbances and medication side effects may contribute independently,^23^ indicating that fatigue requires a multimodal management approach beyond immunosuppression.

Anifrolumab was generally well tolerated with high drug persistence, in line with trial safety profiles.^13^ However, one patient aged 56 years with underlying congestive heart failure and ischaemic heart disease died in the context of decompensated heart failure and influenza A infection complicated by superinfection and sepsis. The patient was not vaccinated for influenza despite being at high risk of complications. There are concerns regarding viral infections during anifrolumab therapy due to the role of IFN in antiviral immune responses. Available data to date have primarily highlighted an increased risk of herpes zoster reactivation. Trials reported zoster in 5%–9% of anifrolumab patients versus ~2% of placebo.^21^ In our cohort, one of 17 patients (5.9%) developed herpes zoster, a rate consistent with that reported in phase II/III trials. Vaccination should be considered before therapy initiation. Long-term extension trials confirm a favourable safety profile with no unexpected signals over 4 years.^21^ In this small real-world cohort, infectious events were frequent and included one fatal case in a patient with significant cardiovascular comorbidities, highlighting the importance of careful monitoring and vaccination of susceptible patients during treatment.

NETs and LDNs play a central role in driving autoimmunity and IFN-I-related organ damage in patients with SLE.^25^ Elevated NET formation observed in other patient cohorts is thought to contribute to the autoantigen burden and to tissue damage.^26^ In our cohort, levels of NE-DNA and MPO-DNA complexes did not show significant changes over the observation period, consistent with recent evidence indicating that SLE is characterised by accumulation of aggregated cell-free DNA due to impaired NET degradation rather than proportional increases in detectable enzyme-DNA complexes.^10^ Still, the expansion of LDNs at baseline was consistent with ongoing neutrophil dysfunction. Importantly, we observed a significant reduction in the proportion of LDNs towards the end of follow-up, suggesting that anifrolumab interferes with the IFN-I-driven inflammatory amplification loop. Similar reductions in LDNs have previously been reported with conventional therapies such as hydroxychloroquine and have been linked to disease activity and progression.^27^ In parallel, anifrolumab markedly downregulated IFN-driven chemokines, including IP-10 and I-TAC, consistent with findings from a large phase III longitudinal proteomics analysis.^28^ TNF-α was also among the cytokines most strongly downmodulated by treatment. Taken together, these findings indicate a broad suppression of chemokine and cytokine networks by anifrolumab, accompanied by a reduction in circulating LDNs and phenotypic changes within this subset. However, because NET-associated serum markers remained stable, our data do not support a definitive conclusion regarding inhibition of NETosis.

The limitations of this study include the small sample size, the absence of a control group and the relatively low prevalence of certain clinical manifestations, including constitutional and haematological features. In addition, patients with nephritis or CNS involvement were not included, which may limit the generalisability of our findings. Moreover, correlation analyses between cytokine changes and clinical outcomes were not performed, as these would have been substantially underpowered due to the small sample size. Nevertheless, the consistency of our findings with randomised controlled trials, the real-world multicentre setting, the downregulation of IFN-associated and neutrophil-related mediators, together with the depth of immunological phenotyping, strengthen the validity and relevance of our observations. Larger multicentre real-world cohorts and registry data will be essential to confirm and extend these findings.

This longitudinal real-world study provides clinical and mechanistic evidence that anifrolumab can meaningfully improve clinical outcomes and facilitate glucocorticoid tapering in patients with active SLE by targeting the IFN-I pathway and reducing the frequency of circulating LDNs, with associated phenotypic changes in this neutrophil subset. After 1 year of therapy, the majority of patients achieved low disease activity, with substantial reductions in global disease indices, joint and skin involvement and improvements in haematological parameters. The effects of anifrolumab appear particularly relevant for patients with IFN-driven disease, such as those with cutaneous manifestations or a pronounced IFN signature. In this real-world cohort, anifrolumab achieved high effectiveness with favourable tolerability. Nevertheless, complete and persistent remission remains uncommon, occurring in only about one-quarter of patients in cohort studies.^3^ Key open questions include optimal integration with existing therapies, treatment duration and long-term effects on outcomes such as organ damage and mortality. Ongoing real-world registries, including Anifrolumab Study for Treatment Effectiveness in the Real World,^29^ and further research into predictive biomarkers will be essential to refine patient selection and define the optimal use of IFN blockade in SLE.

## Supplementary material

10.1136/rmdopen-2026-006806 online supplemental file 1

## Data Availability

Data are available on reasonable request.
