# Supplementary material for: Real-world longitudinal assessment of anifrolumab in patients with systemic lupus erythematosus: clinical outcomes, safety and modulation of cytokines and neutrophil activity
Source: RMD Open. 2026 Jun 28;12(2):e006806. doi: 10.1136/rmdopen-2026-006806 (PMC13331157; doi:10.1136/rmdopen-2026-006806)
Supplement: online supplemental file 1 [file rmdopen-12-2-s001.docx]

**Supplementary Table 1:** Baseline clinical characteristics and longitudinal changes in clinical, laboratory, and patient-reported outcomes.

| **Outcome** | **Baseline** | **Month 3** | **Month 6** | **Month 9** | **Month 12** | **p (0-3)** | **p (0-6)** | **p (0-9)** | **p (0-12)** |
| --- | --- | --- | --- | --- | --- | --- | --- | --- | --- |
|  | **N=20** | **N=17** | **N=17** | **N=17** | **N=13** |  |  |  |  |
| **Clinical parameters** | | | | | | | | | |
| SLEDAI: CNS | 0 (0-0) | 0 (0-0) | 0 (0-0) | 0 (0-0) | 0 (0-0) | NA | NA | NA | NA |
| SLEDAI: Vasculitis | 1.62 (0.5-2.7) | 0.83 (0-1.7) | 0.34 (0-1.3) | 0.21 (0-1.1) | 0.3 (0-1.5) | 0.133 | 0.063 | **0.029** | **0.049** |
| SLEDAI: Renal | 0.68 (0-1.5) | 0.61 (0-1.3) | 0.58 (0-1.4) | 0.68 (0-1.5) | 0.91 (0-1.9) | 0.805 | 0.832 | 0.986 | 0.566 |
| SLEDAI: Musculoskeletal | 2.9 (2-3.8) | 1.72 (0.8-2.6) | 1.2 (0.4-2) | 1 (0.2-1.9) | 0.62 (0-1.5) | **0.014** | **<0.001** | **<0.001** | **<0.001** |
| SLEDAI: Skin | 2.32 (1.6-3) | 0.98 (0.4-1.6) | 0.48 (0-1) | 0.7 (0.1-1.3) | 1.11 (0.4-1.8) | **0.001** | **<0.001** | **<0.001** | **0.011** |
| SLEDAI: Serositis | 0 (0-0) | 0 (0-0) | 0 (0-0) | 0 (0-0) | 0 (0-0) | NA | NA | NA | NA |
| SLEDAI: Hematological | 0.17 (0-1) | 0 (0-1) | 0 (0-1) | 0 (0-1) | 0 (0-1) | 0.316 | 0.316 | 0.316 | 0.316 |
| SLEDAI: Constitutional | 0 (0-0) | 0 (0-0) | 0 (0-0) | 0 (0-0) | 0 (0-0) | NA | NA | NA | NA |
| SLEDAI: Immunological | 2.46 (1.8-3.1) | 2.29 (1.6-3) | 2.13 (1.5-2.8) | 1.97 (1.3-2.6) | 1.8 (1.1-2.5) | **0.015** | **0.015** | **0.015** | **0.015** |
| SLEDAI: Total Score | 11 (9-13) | 6.07 (4.2-7.9) | 4.52 (2.8-6.3) | 4.38 (2.5-6.2) | 3.1 (1.1-5.1) | **<0.001** | **<0.001** | **<0.001** | **<0.001** |
| SLEDAI Response (SRI-4) | 0 (0-0.5) | 0.64 (0.1-1) | 0.92 (0.3-1) | 0.89 (0.2-1) | 0.97 (0.3-1) | **0.017** | **0.010** | **0.006** | **0.009** |
| LLDAS | 0 (0-0.2) | 0.08 (0-0.3) | 0.27 (0.1-0.6) | 0.6 (0.3-0.9) | 0.86 (0.5-1) | **<0.001** | **<0.001** | **<0.001** | **<0.001** |
| DORIS (Remission) | 0 (0-0.1) | 0.02 (0-0.2) | 0.14 (0-0.4) | 0.53 (0.3-0.8) | 0.89 (0.6-1) | **<0.001** | **<0.001** | **<0.001** | **<0.001** |
| Physician global assessment (PGA) | 1.57 (1.3-1.9) | 1.19 (0.9-1.5) | 0.83 (0.6-1.1) | 0.49 (0.2-0.7) | 0.16 (0-0.5) | **<0.001** | **<0.001** | **<0.001** | **<0.001** |
| SJC | 1.37 (0.1-2.7) | 1.29 (0-2.6) | 1.22 (0-2.5) | 1.14 (0-2.4) | 1.06 (0-2.4) | 0.262 | 0.261 | 0.261 | 0.261 |
| TJC | 3.92 (1.6-6.2) | 2.36 (0.3-4.4) | 1.54 (0-3.5) | 1.24 (0-3.2) | 0.95 (0-3.2) | 0.181 | 0.056 | **0.012** | **0.009** |
| DAS28-CRP | 3.12 (2.5-3.8) | 2.93 (2.3-3.6) | 2.75 (2.1-3.4) | 2.57 (1.9-3.2) | 2.39 (1.7-3) | **<0.001** | **<0.001** | **<0.001** | **<0.001** |
| **Patient-reported outcomes** | | | | | | | | | |
| Pain (Patient-rated) | 3.98 (2.6-5.4) | 3.76 (2.4-5.1) | 3.54 (2.2-4.9) | 3.32 (1.9-4.7) | 3.1 (1.7-4.5) | **0.042** | **0.042** | **0.042** | **0.042** |
| Global Activity (Patient-rated) | 4.33 (2.9-5.7) | 3.83 (2.5-5.2) | 3.5 (2.2-4.8) | 3.31 (2-4.6) | 3.18 (1.7-4.6) | 0.222 | 0.104 | **0.028** | **0.014** |
| HAQ | 1.24 (0.8-1.7) | 1.09 (0.7-1.5) | 1 (0.6-1.4) | 1 (0.6-1.4) | 1.08 (0.6-1.5) | 0.148 | 0.087 | 0.081 | 0.254 |
| FACIT-Fatigue | 24 (15-33) | 25 (16-34) | 25 (16-34) | 24 (16-33) | 23 (14-32) | 0.449 | 0.510 | 0.907 | 0.524 |
| **Laboratory parameters** | | | | | | | | | |
| Hemoglobin (g/dL) | 12 (12-13) | 12 (12-13) | 13 (12-13) | 13 (12-13) | 13 (12-14) | **0.008** | **0.008** | **0.008** | **0.008** |
| Leukocytes (10³/µL) | 6.05 (4.6-7.5) | 6.39 (5-7.8) | 6.74 (5.3-8.1) | 7.08 (5.7-8.5) | 7.43 (5.9-8.9) | **0.004** | **0.004** | **0.004** | **0.004** |
| Neutrophils (%) | 63 (58-67) | 64 (60-68) | 65 (61-69) | 66 (62-70) | 67 (63-72) | 0.096 | 0.096 | 0.096 | 0.096 |
| Lymphocytes (%) | 24 (20-28) | 23 (20-26) | 23 (20-26) | 22 (20-25) | 22 (18-26) | 0.485 | 0.485 | 0.484 | 0.484 |
| Platelets (10³/µL) | 268 (213-323) | 278 (224-332) | 289 (235-342) | 299 (245-353) | 309 (254-365) | **0.002** | **0.002** | **0.002** | **0.002** |
| CRP (mg/L) | 12 (8-15) | 11 (8-14) | 9.82 (7.3-12.3) | 8.91 (6-11.8) | 8 (4.1-11.9) | 0.230 | 0.228 | 0.224 | 0.222 |
| ESR (mm/h) | 14 (7-22) | 15 (9-22) | 16 (11-22) | 18 (11-24) | 19 (11-26) | 0.389 | 0.388 | 0.388 | 0.387 |
| ds-DNA (IU/mL) | 240 (72-407) | 225 (70-380) | 211 (59-362) | 196 (38-353) | 181 (9-354) | 0.459 | 0.459 | 0.458 | 0.457 |
| Complement C3 (mg/dL) | 90 (73-107) | 91 (74-108) | 91 (75-108) | 92 (75-109) | 93 (76-110) | 0.219 | 0.218 | 0.218 | 0.217 |
| Complement C4 (mg/dL) | 16 (12-19) | 15 (11-19) | 15 (11-18) | 14 (10-18) | 13 (10-17) | **<0.001** | **<0.001** | **<0.001** | **<0.001** |
| Values are estimated marginal means with 95% confidence intervals from generalized additive models. P-values reflect model-based contrasts between baseline and follow-up visits at months 3, 6, 9, and 12.  Abbreviations: SJC, swollen joint count; TJC, tender joint count; FACIT-Fatigue, Functional Assessment of Chronic Illness Therapy-Fatigue; HAQ, Health Assessment Questionnaire Disability Index; LLDAS, Lupus Low Disease Activity State; SLEDAI, Systemic Lupus Erythematosus Disease Activity Index; DAS28-CRP, 28-joint Disease Activity Score based on C-reactive protein; CRP, C-reactive protein; ESR, erythrocyte sedimentation rate; LDN, low-density neutrophils. | | | | | | | | | |

**Supplementary Table 2:** Longitudinal changes in cytokines and neutrophil extracellular traps parameters

| **Outcome** | **Baseline** | **Month 3** | **Month 6** | **Month 9** | **Month 12** | **p (0-3)** | **p (0-6)** | **p (0-9)** | **p (0-12)** |
| --- | --- | --- | --- | --- | --- | --- | --- | --- | --- |
| **Low-density Neutrophils and NETs** | | | | | | | | | |
| **LDN (%)** | 5.65 (2.3-9) | 3.75 (1.9-5.6) | 2.3 (0.2-4.4) | 1.5 (0-3.5) | 1.16 (0-3.1) | 0.218 | 0.143 | 0.075 | **0.037** |
| **MPO-DNA** | 0.09 (0-0.2) | 0.1 (0-0.2) | 0.12 (0.1-0.2) | 0.14 (0.1-0.2) | 0.15 (0.1-0.2) | 0.278 | 0.231 | 0.170 | 0.128 |
| **NE-DNA** | 0.12 (0-0.2) | 0.14 (0.1-0.2) | 0.16 (0.1-0.2) | 0.18 (0.1-0.3) | 0.19 (0.1-0.3) | 0.425 | 0.391 | 0.327 | 0.252 |
| **Neutrophil chemokines** | | | | | | | | | |
| **RANTES** | 26 (21-30) | 24 (20-28) | 22 (18-26) | 20 (16-25) | 19 (14-23) | **<0.001** | **<0.001** | **<0.001** | **<0.001** |
| **MCP-1** | 1218 (536-1900) | 1136 (468-1803) | 1054 (394-1713) | 972 (313-1630) | 889 (224-1554) | **0.018** | **0.018** | **0.018** | **0.018** |
| **TARC** | 3118 (2241-3996) | 3045 (2317-3773) | 2972 (2337-3607) | 2898 (2288-3508) | 2824 (2169-3480) | 0.556 | 0.547 | 0.533 | 0.522 |
| **Eotaxin** | 167 (85-249) | 154 (75-234) | 141 (63-219) | 129 (51-206) | 116 (37-195) | **0.010** | **0.010** | **0.010** | **0.010** |
| **IP-10** | 4.91 (3.8-6) | 4.64 (3.6-5.7) | 4.38 (3.4-5.4) | 4.11 (3.1-5.1) | 3.85 (2.8-4.9) | **<0.001** | **<0.001** | **<0.001** | **<0.001** |
| **IL-8** | 20 (16-25) | 19 (14-23) | 17 (13-21) | 15 (11-19) | 13 (9-17) | **<0.001** | **<0.001** | **<0.001** | **<0.001** |
| **MIP-1β** | 12 (10-14) | 11 (10-13) | 11 (9-12) | 9.71 (8.1-11.3) | 8.89 (7.3-10.5) | **<0.001** | **<0.001** | **<0.001** | **<0.001** |
| **I-TAC** | 11 (9-14) | 11 (8-13) | 9.74 (7.6-11.8) | 8.98 (6.9-11.1) | 8.22 (6.1-10.4) | **<0.001** | **<0.001** | **<0.001** | **<0.001** |
| **GRO-α** | 3862 (0-13343) | 4163 (0-12840) | 4464 (0-12712) | 4762 (0-12966) | 5057 (0-13565) | 0.761 | 0.758 | 0.753 | 0.749 |
| **MIP-3α** | 8.12 (6.8-9.4) | 7.47 (6.4-8.5) | 6.84 (5.8-7.9) | 6.26 (5.1-7.4) | 5.75 (4.6-6.9) | **0.022** | **0.013** | **0.004** | **<0.001** |
| **ENA-78** | 4.35 (3.3-5.3) | 4.12 (3.1-5.1) | 3.9 (2.9-4.9) | 3.67 (2.7-4.6) | 3.45 (2.5-4.4) | **<0.001** | **<0.001** | **<0.001** | **<0.001** |
| **Cytokines** | | | | | | | | | |
| **IL-1β** | 115 (108-123) | 115 (108-122) | 115 (108-122) | 115 (108-122) | 115 (108-122) | 0.977 | 0.977 | 0.977 | 0.977 |
| **IL-6** | 85 (78-92) | 84 (78-91) | 83 (77-89) | 83 (77-89) | 82 (76-88) | 0.234 | 0.234 | 0.234 | 0.234 |
| **TNF-α** | 229 (187-272) | 210 (179-240) | 191 (158-223) | 175 (139-210) | 162 (128-197) | 0.119 | 0.089 | **0.046** | **0.014** |
| **IFN-α2** | 48 (44-53) | 49 (45-53) | 49 (45-53) | 49 (45-53) | 49 (45-53) | 0.596 | 0.596 | 0.595 | 0.595 |
| **IFN-γ** | 147 (120-174) | 147 (123-171) | 146 (123-168) | 145 (123-168) | 145 (121-169) | 0.847 | 0.847 | 0.846 | 0.846 |
| **IL-12p70** | 53 (50-56) | 53 (50-55) | 52 (50-55) | 52 (49-55) | 52 (49-54) | 0.353 | 0.353 | 0.353 | 0.352 |
| **IL-17A** | 16 (14-17) | 16 (15-17) | 16 (15-16) | 16 (15-16) | 16 (15-17) | 0.963 | 0.963 | 0.963 | 0.963 |
| **IL-18** | 522 (456-587) | 523 (463-583) | 524 (467-581) | 525 (468-582) | 526 (467-586) | 0.866 | 0.866 | 0.866 | 0.866 |
| **IL-23** | 142 (131-152) | 143 (133-153) | 145 (135-154) | 146 (137-156) | 148 (138-157) | 0.122 | 0.122 | 0.122 | 0.122 |
| **IL-10** | 38 (32-43) | 39 (35-42) | 39 (35-44) | 39 (34-43) | 37 (33-41) | 0.659 | 0.674 | 0.810 | 0.814 |
| **IL-33** | 587 (547-626) | 588 (551-625) | 590 (554-626) | 591 (555-627) | 593 (556-630) | 0.612 | 0.612 | 0.612 | 0.611 |
| Values are estimated marginal means with 95% confidence intervals from generalized additive models. P-values reflect model-based contrasts between baseline and follow-up visits at months 3, 6, 9, and 12. Outcomes include circulating cytokines, chemokines, and NET-associated markers measured longitudinally to characterize immunologic changes over the 12-month follow-up.  Abbreviations: MPO-DNA, myeloperoxidase-DNA complexes; NE-DNA, neutrophil elastase-DNA complexes; RANTES, regulated upon activation normal T cell expressed and secreted (CCL5); MCP-1, monocyte chemoattractant protein 1 (CCL2); TARC, thymus and activation-regulated chemokine (CCL17); IP- 10, interferon gamma-induced protein 10 (CXCL10); IL, interleukin; IFN, interferon; TNF, tumor necrosis factor; I-TAC, interferon-inducible T-cell alpha chemoattractant (CXCL11); GRO-α, growth-related oncogene alpha (CXCL1); MIP-1β, macrophage inflammatory protein 1 beta (CCL4); MIP-3α, macrophage inflammatory protein 3 alpha (CCL20); ENA-78, epithelial neutrophil-activating peptide 78 (CXCL5). | | | | | | | | | |


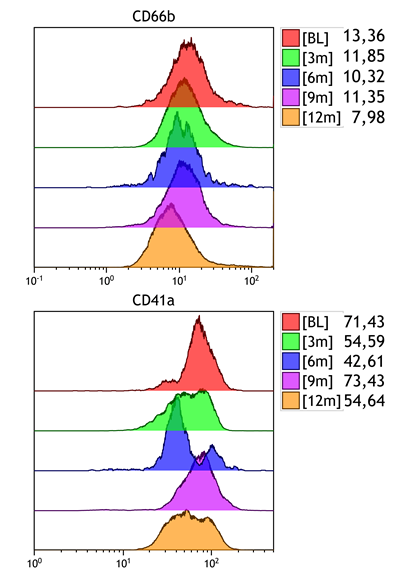


**Supplementary Figure 1.**

Composite offset histograms and median fluorescence intensity of low-density neutrophils by time point for the activation markers CD66b and CD41a.
